# Asymmetric Fractal Characteristics and Market Efficiency Analysis of Style Stock Indices

**DOI:** 10.3390/e24070969

**Published:** 2022-07-13

**Authors:** Chao Xu, Jinchuan Ke, Zhikai Peng, Wen Fang, Yu Duan

**Affiliations:** School of Economics and Management, Beijing Jiaotong University, Beijing 100044, China; 18113017@bjtu.edu.cn (C.X.); 17113130@bjtu.edu.cn (Z.P.); wenfang@bjtu.edu.cn (W.F.); 18113003@bjtu.edu.cn (Y.D.)

**Keywords:** style stock indices, multifractals, asymmetry, market efficiency

## Abstract

As a typical complex system, the stock market has attracted the attention of scholars and investors to comprehensively understand its fractal characteristics and analyze its market efficiency. Firstly, this paper proposes an asymmetric, detrended fluctuation analysis based on overlapping sliding windows (OSW-A-MFDFA). It reduces the generation of fluctuation errors, and the calculation results are more robust and reliable. The advantage of the OSW-A-MFDFA is that it not only can reveal the multifractal characteristics of time series clearly, but also can further accurately analyze the asymmetry of fractal characteristics under different trends. Secondly, this paper focuses on the variation in the width difference and height difference of the multifractal spectrum under different trends. Finally, based on multifractality, this paper proposes a comprehensive indicator MED that can be used to measure market efficiency, which is characterized by traversing all fluctuation orders. The application revealed many interesting findings in style stock indices. Style stock indices have asymmetric multifractal characteristics, and there are significant differences in the fractal spectrum of different style assets. Moreover, the market efficiency of style stock indices is time-varying, which can be reasonably explained from the perspective of the adaptive market hypothesis.

## 1. Introduction

It is well known that volatility in financial markets and market efficiency are enduring topics of discussion [1]. The research on it not only aroused the interest of the majority of scholars, but also attracted the attention of the practical circle [2]. In fact, analyzing the volatility characteristics and market efficiency of financial markets is of great significance to both investors and regulators. For the majority of investors, if they can accurately judge the fluctuation trend in financial markets, they can adjust their investment strategies in a timely manner in order to further improve their investment returns [3]. Similarly, accurate analysis of volatility characteristics and market efficiency can help regulators to work more scientifically. On the one hand, regulators can focus on the supervision of assets that may have volatility trend transformation in the future, and take relevant measures to avoid abnormal market volatility. On the other hand, regulators can timely intervene when markets are inefficient to ensure the healthy development of financial markets. In addition, the effectiveness of financial markets will directly affect the effective flow of spare funds, and then affect the effective allocation of resources in the real economy. It can be seen that an accurate and comprehensive discussion of the volatility characteristics and efficiency of financial markets is imminent.

In earlier studies, the analysis of financial markets was usually based on the efficient market hypothesis (EMH) [4]. In the modern mainstream financial theory, the capital asset pricing model, option pricing theory, arbitrage pricing theory and other classic capital market theories are all like this [5]. They all believe that the market is efficient. There are several basic assumptions in efficient market theory, such as investors’ perfect rationality, investors’ risk aversion and perfect market competition. From these assumptions, efficient market theory argues that capital markets are efficient. The EMH holds that in a stock market with sound laws, high transparency and full competition, all valuable information has been reflected in stock prices in a timely and accurate manner [6]. When the financial market is an efficient market, the price movements of financial instruments are completely random and follow a random walk process. The autocorrelation function of the difference term of the random walk process is 0, which has no memory. In an efficient market, investors cannot obtain excess returns based on the information they have, and the price trends in financial instruments are difficult to predict. Because the price at every moment is a complete response to market information, the price series does not exhibit significant memory. Many studies have confirmed that the Hurst index of completely independent random walk sequences should be equal to 0.5 [7]. Therefore, the Hurst index calculated by the price series of financial instruments under the EMH should not deviate significantly from 0.5.

However, a large number of financial anomalies continue to challenge the EMH theory, such as the long memory of financial markets [8]. With the in-depth follow-up research, many research results show that the real financial market is far from the efficient market [9]. Many scholars have found that financial markets have fractal nonlinear characteristics, which indicates that financial markets do not conform to the EMH [10,11]. The fractal market hypothesis (FMH) came into being, which provides a new perspective for the study of financial markets. Fractal market theory breaks the simple and linear way of thinking in efficient market theory and challenges the foundations of mainstream financial theory. In the fractal market theory proposed by Peters, he believes that the assumptions of efficient market theory for investors are too simplistic. Investors are not completely rational in real capital markets; they are more likely to be bounded rationality [12].

Fractal market theory believes that the movement of asset prices can be described by geometric fractional Brownian motion. When the price of an asset follows the geometric fractal Brownian motion, the return on the asset follows the fractal Brownian motion. When the Hurst value is equal to 0.5, the fractal Brownian motion degenerates into standard Brownian motion, and asset prices obey geometric Brownian motion. The autocovariance function of the return on assets series is 0 at the moment. This shows that there is no correlation in the return on assets series. The sequences of returns are independent and completely random. At this time, the financial market is an efficient market, because current information does not affect the future, and asset prices have fully reflected current information. When the Hurst value is greater than 0 and less than 0.5, the autocovariance function of the asset return series converges to a negative number asymptotically. This shows that there is a negative correlation between the current return and a return over a longer period of time. The return series is an antipersistence series. When the sequence was moving up at the previous moment, then the sequence is likely to be moving down at the next moment. Such time series are more deformable than completely random series. Conversely, when the Hurst value is between 0.5 and 1, the autocovariance function of the asset return series asymptotically converges to a positive number. This shows that there is a positive correlation between the current return and a return over a longer period of time. The return series is a persistent series or trend-enhancing series. When the sequence moves upward at the previous moment, it is likely that the sequence at the next moment is also moving upward. Such phenomena indicate that asset prices do not fully reflect current information and that markets are not fully efficient. In fact, the Hurst index analyzes whether the market is efficient by measuring how well asset prices reflect historical information. However, perhaps behavioral finance can explain the deviation in the Hurst index. This may be related to the irrational investment behaviors such as blindly following the trend and chasing up and down in the market.

When applying fractal theory to the financial market, scholars initially only considered the case where the market has a single-fractal structure. The single fractal only uses the single Hurst index to judge the fractal structure of the market, but this method can only describe the overall overview of the market price changes, and cannot accurately describe its local characteristics. However, the fractal structure of stock price series is not invariable in reality. When the price movement of the market has local violent fluctuations, the single Hurst index will ignore this phenomenon. A single Hurst index can only describe the overall characteristics, and will lose a lot of local fine structure information, so it cannot accurately and truly reflect the characteristics of market price changes. In contrast, multifractal theory can describe the price changes in complex systems from both local and global aspects. At present, FMH has been supported by numerous academic research and praised by some scholars as a powerful tool to study financial markets. In addition, FMH has also achieved excellent results in the practice of portfolio optimization and financial risk warning [13]. As an important representative of the financial market, the stock market must have fractal characteristics in its price fluctuations. In view of this, it is extremely crucial to fully understand the fractal characteristics of the stock market and to reasonably analyze the market efficiency on this basis.

The differences from previous studies are mainly reflected in the methodology and research objects. From the perspective of methodology, this paper applies asymmetric multifractal detrended fluctuation analysis (A-MFDFA) instead of traditional multifractal detrended fluctuation analysis (MFDFA). Its advantage is that it can not only reveal whether the fractal characteristic is single fractal or multifractal, but also analyze the asymmetry of the fractal characteristics. Moreover, this paper improves the original method and constructs an asymmetric multifractal detrended fluctuation analysis based on overlapping sliding windows (OSW-A-MFDFA). The calculation results of the improved method will be more robust and reliable. Furthermore, this paper proposes a new market efficiency indicator under the multifractal paradigm. Its characteristic lies in that it can traverse all the fluctuation orders, which satisfies the reality of multifractal. From the point of view of the research object, this paper is also different from the previous research. Previous studies have mainly focused on the S&P 500, HSI and other comprehensive stock indices in the stock market, but little attention has been paid to style stock indices. Style stock index refers to the index reflecting a particular style or investment characteristics in the market, which is of great reference value to the design of investment portfolio. For example, large-cap stock, mid-cap stock and small-cap stock are differentiated by equity scale; high-price stock, mid-price and penny stocks are differentiated by share price; blue-chip stock, low-profit stock and losing stock are differentiated by performance. However, this article focuses primarily on this type of style indices in the stock market. Style stock indices are built on a specific investment purpose or preference, and they are created to beat the market. Style indices may have different characteristics than composite indices. Furthermore, the market efficiency presented by the style stock indices may also be different from that of the composite stock indices. Style stock indices can be inefficient and may generate excess returns for investors. Therefore, it is of great significance to study the characteristics of style stock indices.

Taking the three types of style indices related to equity scale, share price and performance as research samples, some interesting findings can be obtained in this paper. Firstly, the fractal characteristics of stock market style indices are multiple and asymmetric. In other words, there are distinct differences in fractal characteristics under upward, downward and overall trends. Secondly, the width difference and height difference of multifractal spectrum of assets with different styles are obviously different. Thirdly, the market efficiency of different styles of assets is time-varying. The effectiveness of stock market in crisis periods is in a continuous downturn. The time-varying characteristics can be reasonably explained from the perspective of adaptive market hypothesis (AMH). The research in this paper reflects the characteristics of different styles of assets and brings certain reference value to investors’ investment behavior.

The rest of this paper is organized as follows. Section 2 provides an overview of the relevant literature. The research methods are introduced in Section 3. Data description and descriptive statistical results are given in Section 4. Section 5 shows the empirical analysis results in detail. A brief conclusion is shown in Section 6.

## 2. Literature Review

In recent years, many international scholars have applied a variety of methods and models to study the fractal characteristics of financial markets and evaluate market efficiency. Specifically, the characterization methods of fractal characteristics can be summarized from two aspects: single fractal and multifractal.

Early studies mainly focused on single fractals, including the classical rescale range method (R/S), the modified R/S method proposed by Lo (1991), and the detrended fluctuation analysis method (DFA) proposed by Peng (1994), etc. [14,15,16]. For example, some scholars have found that there are obvious fractal nonlinear characteristics in both the Shanghai securities market and the Shenzhen securities market by means of the R/S method [17]. In addition, many scholars took stock markets of different countries as research objects and used the R/S method to describe their fractal characteristics [18,19,20]. The empirical results all prove that the stock markets of different countries are not the efficient markets mentioned in the EMH, but the markets with typical fractal nonlinear characteristics. Although the R/S method can describe the fractal characteristics of each stock market, the premise of the application of the R/S method is that the time series it targets must be a stationary time series. In fact, a large number of studies have proved that financial time series are often nonstationary [21,22]. In addition, the Hurst index calculated by R/S method is prone to the interference of short-term memory, so the calculation results are often higher than the actual situation.

In view of the limitations of R/S in describing the fractal characteristics of the stock market, the DFA came into being, which makes the characterization of the fractal characteristics of the stock market more scientific and reasonable [23,24]. It can directly model and analyze nonstationary time series, so it is favored by many scholars, and is widely used in the characterization of fractal characteristics of the stock market [25,26]. However, it should be pointed out that both R/S analysis and DFA belong to the research methods of the single-fractal theory. Unfortunately, they can only describe the fractal characteristics of financial time series on the whole, but cannot observe the fractal characteristics of financial time series from a local perspective.

The research on multifractality is a recent research hotspot, and the main analysis methods include the MFDFA method and wavelet analysis method [27,28,29,30]. In addition, the multifractal model of asset returns (MMAR) and the Markov-switching multifractal model (MSM) are also widely used tools to characterize asset volatility in recent years. In particular, Liu et al. (2008) pointed out that the use of scale estimation and Legendre transform methods in statistical physics to estimate the parameters of MMAR is inaccurate [31]. The problems of MSM mainly appear in the difficulty of modeling, the lack of clear standards for algorithm selection, and the complexity of estimation methods. In comparison, the MFDFA method, which does not require too much consideration of the parameter estimation problem, is the most widely used. In order to overcome the shortcomings of the R/S analysis and the DFA, Kantelhardt improved the DFA method and proposed the MFDFA to describe the multifractal characteristics of time series [32]. It can fully display the fractal structure of financial time series both from the whole and from the part. Therefore, compared with the R/S and the DFA, the advantages of the MFDFA are obvious. It is precisely because of its outstanding advantages that MFDFA has been continuously applied by scholars in the research of various disciplines once it was proposed. There is overwhelming evidence that DFA-based extension methods are the dominant method in fractal characteristic research today [33,34].

Many achievements have emerged in the application of the MFDFA in the stock market [35]. Cai and Hong used the MFDFA to investigate the correlation between stock market trading volume and investor fear index. The empirical results show that the dynamic relationship between the volatility of stock market trading volume and different types of investor fear indicators has the characteristics of multifractal, and the dynamic relationship between them has a strong antipersistence [36]. With the application of the MFDFA, some scholars have found that the stock markets of China and the United States both have multifractal characteristics before and after the outbreak of COVID-19. Furthermore, they analyzed the causes of multifractal characteristics, and their research believed that the long memory was the main source of the stock market multifractal characteristics [37]. Some scholars have used the MFDFA based on the generalized Hurst index to compare the relative efficiency of stock markets in developed and emerging countries. The research reveals that most stock markets are multifractal and the long-term efficiency is higher than the short-term efficiency [38]. Moreover, the degree of multifractality in emerging stock markets is higher than that in developed stock markets. The reason for this may be related to the imperfect economic system of the emerging stock market, insufficient financial supervision and a stronger speculative atmosphere. Some scholars combine the Hurst index with the weighted moving time window for dynamic calculation to evaluate the stability level of financial companies [39]. The results show that the multifractal characteristics intensify during the crisis, which suggests that the multifractal characteristics of the time series is changing.

However, financial markets typically exhibit both upward and downward market states. In order to obtain more valuable information for investment portfolios and risk warnings, it is reasonable to analyze the multifractal characteristics separately in these two market states. Traditional DFA and the MFDFA cannot reveal the asymmetry of fractal characteristics. In this case, some scholars have extended the original DFA to an asymmetric DFA to explore the single-fractal characteristics of price series under different trends [40]. Subsequently, the asymmetric MFDFA method (A-MFDFA) that can explore the asymmetric characteristics of multifractals has also emerged. This approach is seen as an extension of MFDFA, which is advanced in its ability to detect the presence of asymmetric multifractal characteristics in complex signal sequences. Correspondingly, many scholars have also confirmed the existence of asymmetric effects in the capital market [41]. Their research results show that the degree of volatility under different trends is different, and the degree of market volatility in a downtrend is often greater than that in an uptrend [42,43]. In view of this, more and more studies use asymmetric fractal characterization methods to test the long memory of various capital markets [44,45,46].

In particular, based on the understanding of fractal characteristics, market efficiency has attracted the attention of scholars [47]. The famous scholar Peters also pointed out in his work that the Hurst index is related to the series of returns. When there is a correlation in the asset return series, investors may predict changes in asset prices in certain ways. This means that asset prices do not fully reflect the historical information associated with them, so Hurst can be used to indirectly measure the efficiency of the market [48]. The Hurst index actually measures how well asset prices reflect historical information. Previous studies have basically reached a consensus. When the Hurst value is 0.5, the correlation of return on assets series is 0, and the change in asset price is completely random. At this time, the performance of the capital market is consistent with the market performance under the efficient market theory, and investors cannot predict changes in asset prices at all. Historical information about an asset is immediately reflected in the asset’s market price, and the market is most efficient at this time. The closer Hurst is to 0.5, the weaker the correlation between asset returns. The more random the asset price movement is, the less likely it is that investors will somehow predict the asset price movement. The shorter the time lag that the historical information about the asset is reflected in the asset market price, the more efficient the market is [49]. Conversely, the more Hurst deviates from 0.5, the stronger the correlation of asset returns. The longer the time lag that the historical information about the asset is reflected in the asset market price, the less efficient the market is. In addition, much of the literature has analyzed the reasons for the deviation in the Hurst index from 0.5 from the perspective of behavioral finance, and studies have shown that this may be related to herd behavior [50]. Some irrational investment behaviors prevail in the long term, such as follow the trend blindly and chase sell [51].

## 3. Method

### 3.1. The A-MFDFA Method and Improvement

The advantage of DFA series methods is that they can effectively exclude the spurious long-range correlation caused by the nonstationarity of time series, so as to truly reveal the part of long-range correlation that represents the dynamic behavior of complex system. Moreover, the MFDFA can comprehensively show the fractal structure of financial time series from both the whole and the part. The fly in the ointment is that the traditional multifractal theory can only identify multifractal characteristics, but cannot capture the fractal characteristics of series on different trends. Here, we introduce A-MFDFA to further study the multifractal properties of financial time series. It retains the advantages of the MFDFA, and can further observe the asymmetry of fractal characteristics.

Let $\left\{x\left(t\right)\right\}$ be financial time series, t=1,2,⋯,N, where N is the length of the time series. The specific operation steps of the A-MFDFA are as follows:

Step 1: We construct the profile of the initial financial time series $y\left(j\right)=\overset{j}{\underset{t=1}{\sum }}\left(x\left(t\right)-\bar{x}\right)$, j=1,2,⋯,N. Here, $\bar{x}$ is the mean of $x\left(t\right)$.

Step 2: The original time series $x\left(t\right)$ and its profile $y\left(j\right)$ are divided into $N_{n}$ nonoverlapping subcontinuous segments of equal length n, where $N_{n}=int\left(N/n\right)$. However, the length N of the series is often not an integer multiple of n; that is, tail data redundancy may occur. In order not to lose the last small part of the data, the segmentation process is repeated in reverse order, resulting in a total of $2N_{n}$ segments. Suppose $S_{j}=\left\{s_{j,k},k=1,2,\cdots ,n\right\}$ is the j-th sub-time series of length n of $\left\{x\left(t\right)\right\}$, $Y_{j}=\left\{y_{j,k},k=1,2,\cdots ,n\right\}$ is the j-th sub-time series of length n of $\left\{y\left(j\right)\right\}$, and $j=1,2,\cdots ,2N_{n}$.

Therefore, for $j=1,2,\cdots ,N_{n}$, there are:

(1) $$s_{j,k}=x\left(\left(j-1\right)n+k\right),y_{j,k}=y\left(\left(j-1\right)n+k\right)$$

for $j=N_{s}+1,\cdots ,2N_{n}$, the following formula holds:

(2) $$s_{j,k}=x\left(N-\left(j-N_{s}\right)n+k\right),\;\;y_{j,k}=y\left(N-\left(j-N_{s}\right)n+k\right)$$

It is worth mentioning that research by Peng et al. (1994) suggested that 5≤n≤N/4 [15].

Step 3: For each sub-time series $S_{j}$ and its profile $Y_{j}$, we characterize the local fluctuation trend by least squares straight line fitting, as follows: $L_{S_{j}}\left(k\right)=a_{S_{j}}+b_{S_{j}}k$ and $L_{Y_{j}}\left(k\right)=a_{Y_{j}}+b_{Y_{j}}k$, where k represents the abscissa. $L_{S_{j}}\left(k\right)$ and $L_{Y_{j}}\left(k\right)$ represent the linear trend in the j-th sub-time series $S_{j}$ and its profile $Y_{j}$, respectively. The linear fit $L_{S_{j}}\left(k\right)$ uses the slope $b_{S_{j}}$ to determine whether the trend in the sub-time series $S_{j}$ is positive or negative. Linear fitting $L_{Y_{j}}\left(k\right)$ is used to disperse the contour time series $Y_{j}$, and define the fluctuation function as $F_{j}\left(n\right)=\frac{1}{n}\sum_{k=1}^{n}{\left(y_{j,k}-L_{Y_{j}}\left(k\right)\right)}^{2}$, $j=1,2,\cdots ,2N_{n}$.

Step 4: To evaluate the asymmetric volatility properties, average volatility functions are constructed when the time series $x\left(t\right)$ has a piecewise positive and negative linear trend. The sign of the slope $b_{S_{j}}$ is used for trend discrimination; that is, $b_{S_{j}}>0$ ($b_{S_{j}}<0$) indicates that the time series $x\left(t\right)$ has a positive (negative) trend. The calculation to define the q-order wave function is as follows:

(3) $$F_{q}^{+}\left(n\right)={\left(\frac{1}{M^{+}}\overset{2N_{n}}{\underset{j=1}{\sum }}\frac{1+sgn\left(b_{S_{j}}\right)}{2}{\left[F_{j}\left(n\right)\right]}^{q/2}\right)}^{1/q}\;$$

(4) $$F_{q}^{-}\left(n\right)={\left(\frac{1}{M^{-}}\overset{2N_{n}}{\underset{j=1}{\sum }}\frac{1-sgn\left(b_{S_{j}}\right)}{2}{\left[F_{j}\left(n\right)\right]}^{q/2}\right)}^{1/q}\;$$

Among them, $F_{q}^{+}\left(n\right)$ and $F_{q}^{-}\left(n\right)$ represent the q-order average fluctuation function of the upward trend and the downward trend, respectively. $M^{+}=\overset{2N_{n}}{\underset{j=1}{\sum }}\frac{1+sgn\left(b_{S_{j}}\right)}{2}$ and $M^{-}=\overset{2N_{n}}{\underset{j=1}{\sum }}\frac{1-sgn\left(b_{S_{j}}\right)}{2}$ represent the number of sub-time series with positive and negative volatility trends, respectively. For all $j=1,2,\cdots ,2N_{n}$, assuming $b_{S_{j}}\neq 0$, then $M^{+}+M^{-}=2N_{n}$.

Step 5: If the time series has long-range correlation, the following power-law relationship can be observed: $F_{q}\left(n\right)\sim n^{H\left(q\right)}$, $F_{q}^{+}\left(n\right)\sim n^{H^{+}\left(q\right)}$ and $F_{q}^{-}\left(n\right)\sim n^{H^{-}\left(q\right)}$. The scaling relationship of the wave function can be determined by analyzing the log–log plots of $F_{q}\left(n\right)$, $F_{q}^{+}\left(n\right)$ and $F_{q}^{-}\left(n\right)$ against each value of q. The least squares method based on logarithmic form can be used to calculate $H\left(q\right)$, $H^{+}\left(q\right)$ and $H^{-}\left(q\right)$.

For the change in Hurst index, we first focus on the following aspects:

(a) If there is no significant change in $H\left(q\right)$ with the change in the fluctuation order q, it indicates that the system exhibits the characteristics of a single fractal. Conversely, if $H\left(q\right)$ changes significantly with the fluctuation order q, it indicates that the system exhibits multifractal characteristics.
(a) For large positive numbers q, $H\left(q\right)$ describes the scaling behavior where large fluctuations dominate. Conversely, for negative or small positive numbers q, $H\left(q\right)$ describes the fractal characteristics dominated by small fluctuations. In particular, when q is equal to 2, $H\left(2\right)$ is the classical Hurst exponent, which measures the overall long memory of the series.
(c) It is worth noting that the correlation in the time series is persistent if $H\left(q\right)>0.5$, which means that one increment is more likely to be followed by another. However, if $H\left(q\right)<0.5$, there is inverse persistence, indicating that an increment is more likely to follow a trend transition. When $H\left(q\right)=0.5$, it means that the sequence follows a random walk process.

In addition, this paper mainly focuses on the asymmetry of fractal characteristics. In particular, if $H^{+}\left(q\right)=H^{-}\left(q\right)$ holds, the correlation between sequences is symmetric. Conversely, if $H^{+}\left(q\right)\neq H^{-}\left(q\right)$ is satisfied, then an asymmetric correlation exists. We define the function $A\left(q\right)$ to measure the degree of asymmetry of the correlation, that is, the degree of difference between $H^{+}\left(q\right)$ and $H^{-}\left(q\right)$, as follows:

(5) $$A\left(q\right)=H^{+}\left(q\right)-H^{-}\left(q\right)$$

For a fixed q, the larger the absolute value of $A\left(q\right)$, the higher the degree of asymmetry of the multifractal characteristics when the trend in the price series is different. If $A\left(q\right)$ is greater than 0, it means that the continuity of the price series in upward trend is higher than that in downward trend. Conversely, if $A\left(q\right)$ is less than 0, it means that when the price series is in downward trend, the price fluctuations are more persistent.

**Figure 1:**
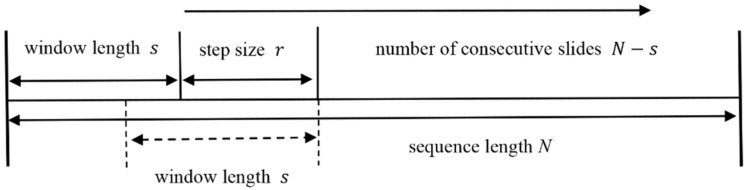
The Improvement in Step 2.

It is worth noting that the traditional A-MFDFA generates new pseudo-fluctuation errors due to the discontinuity of the polynomial fit at the junction points of the split data, causing distortion of the scale index estimates. In order to overcome this deficiency, we improved the data sequence segmentation method in Step 2. Specifically, we use continuous overlapping interval segmentation technology to replace the original segmentation method, as shown in Figure 1. The improved A-MFDFA can greatly reduce the occurrence of pseudo-fluctuation errors. It can not only avoid the error estimation caused by the loss of tail data due to the construction of $N_{n}$ subintervals, but also avoid the error estimation of $H\left(q\right)$ caused by disrupting the order of the original sequence due to the construction of $2N_{n}$ subintervals. This approach results in a jump in the number of subintervals, from $N_{n}$ or $2N_{n}$ to N−s+m, where s is the length of the sliding window and r is the step size for each overlapping window sliding.

In step 4, $M^{+}+M^{-}=N-s+m$, the calculation of the q-order wave function also changes accordingly:

(6) $$F_{q}^{+}\left(n\right)={\left(\frac{1}{M^{+}}\overset{N-s+m}{\underset{j=1}{\sum }}\frac{1+sgn\left(b_{S_{j}}\right)}{2}{\left[F_{j}\left(n\right)\right]}^{q/2}\right)}^{1/q}\;$$

(7) $$F_{q}^{-}\left(n\right)={\left(\frac{1}{M^{-}}\overset{N-s+m}{\underset{j=1}{\sum }}\frac{1-sgn\left(b_{S_{j}}\right)}{2}{\left[F_{j}\left(n\right)\right]}^{q/2}\right)}^{1/q}\;$$

In this way, the improved A-MFDFA can also be called A-MFDFA with overlapping sliding windows (OSW-A-MFDFA). Compared with the original A-MFDFA, the generalized Hurst exponent calculated by it is more accurate and robust.

### 3.2. Multifractal Spectrum

The generalized Hurst exponent $H\left(q\right)$ and the Renyi exponent $\tau \left(q\right)$ obtained by a series of methods such as DFA have the following relationship:

(8) $$\tau \left(q\right)=qH\left(q\right)-1\;$$

Taking the derivative of q on both sides of the above equation, we can obtain

(9) $$\frac{d\tau \left(q\right)}{dq}=H\left(q\right)+qH^{\prime }\left(q\right)\;$$

By Legendre transformation, the relationship among multifractal spectrum $f\left(\alpha \right)$, the singularity strength α and $H\left(q\right)$ can be obtained as follows:

(10) $$\left\{\begin{array}{c} \alpha =H\left(q\right)+qH^{\prime }\left(q\right)\\ f\left(\alpha \right)=q\left[\alpha -H\left(q\right)\right]+1 \end{array}\right.\;$$

Similarly, when $H\left(q\right)$ is replaced by $H^{+}\left(q\right)$ or $H^{-}\left(q\right)$, we will obtain the singularity strength α and multifractal spectrum $f\left(\alpha \right)$ of the time series with an upward trend or a downward trend, respectively.

Two important parameters, namely the width difference dα and the height difference df of the fractal spectrum, can be obtained from the multifractal spectrum $f\left(\alpha \right)$ and singularity strength α. Because α describes the singularity of the time series, the width difference dα of the fractal spectrum uses the difference between the maximum probability and the minimum probability to describe the fluctuation degree and stability of the normalized distribution of the entire series. That is, $d\alpha =\alpha_{max}-\alpha_{min}$. Generally speaking, dα is positively correlated with the volatility of the series. The height difference df can represent the number of occurrences of the calculated singular value, that is, $df=f\left(\alpha_{min}\right)-f\left(\alpha_{max}\right)$. df reflects the ratio of the number of peaks and troughs in the price series. df>0 indicates that the price sequence appears more at the peak position, while df<0 indicates the opposite. Therefore, the shape of the multifractal spectrum can also convey a large amount of information related to volatility risk.

### 3.3. Market Efficiency Indicator

In the EMH, the price of financial instruments will follow a random walk, and its fluctuation trend is difficult to predict. The EMH holds that in a stock market with sound laws, high transparency and full competition, all valuable information has been timely and accurately reflected in stock prices. Unless there is market manipulation, investors cannot obtain excess profits above the market average by analyzing past price information. However, the Hurst index of random walk time series tends not to deviate significantly from 0.5. Therefore, the Hurst value can be used to indirectly characterize the degree of effectiveness of market efficiency. Most studies use the absolute value of the difference between it and 0.5 to measure the degree of market efficiency. Some scholars also obtain an index directly reflecting the relative efficiency of the market by appropriate transformation of Hurst value.

However, when the financial market has multifractal characteristics, its generalized Hurst value will change with the fluctuation order. The generalized Hurst value $H\left(q\right)$ is variable, and so is the market effectiveness represented by it. Wang et al. (2010) proposed the market efficiency index (ED) to measure the market efficiency under q-order volatility in order to take into account different volatility orders in multifractal reality [52]. The larger the value of $ED\left(q\right)$, the further the distance between $H\left(q\right)$ and 0.5, and the lower the market efficiency.

(11) $$ED\left(q\right)=\left|H\left(q\right)-0.5\right|\;$$

Inspired by it, in order to traverse all volatility orders q as a whole, we propose a multifractal-based measure of the degree of market efficiency (MED) to directly measure market efficiency, as follows:

(12) $$MED=1-\frac{1}{q_{max}-q_{min}}\overset{q_{max}}{\underset{q=q_{min}}{\sum }}ED\left(q\right)=1-\frac{1}{q_{max}-q_{min}}\overset{q_{max}}{\underset{q=q_{min}}{\sum }}\left|H\left(q\right)-0.5\right|\;$$

The MED indicator is a comprehensive indicator that includes all fluctuation orders q and $H\left(q\right)$, which takes into account all different kinds of fluctuations. After the calculation of the above formula, we can know that the value range of the market efficiency degree MED is between 0 and 1. The closer the MED value is to 1, the higher the market efficiency. Based on the asymmetry of multifractals, we can also calculate the market efficiency measures under the upward and downward, as follows:

(13) $$MED^{+}=1-\frac{1}{q_{max}-q_{min}}\overset{q_{max}}{\underset{q=q_{min}}{\sum }}\left|H^{+}\left(q\right)-0.5\right|\;$$

(14) $$MED^{-}=1-\frac{1}{q_{max}-q_{min}}\overset{q_{max}}{\underset{q=q_{min}}{\sum }}\left|H^{-}\left(q\right)-0.5\right|\;$$

## 4. Data and Descriptive Statistics

After years of enrichment and improvement, there are more and more types of indices in the stock market. Unlike composite stock indices, style stock indices are indices that reflect a particular style or investment characteristic in the market. Style indices may have different characteristics than composite indices. Furthermore, the market efficiency presented by the style stock indices and the composite stock indices may also be different. This paper selects the Chinese stock market style indices compiled by Shenyin & Wanguo Securities Company (SW style stock indices) for analysis. The style indices are compiled based on factors such as the capital stock size, performance level and stock price level of listed companies. Various style indices are subdivided into three subcategories: large, medium and small (high, medium and low), as shown in Table 1. There are two reasons why the SW style stock indices are selected as the research samples of China’s stock market. One is that the SW style indices were released early, and there are various types of style indices. In addition, the SW style indices are properly compiled and can better reflect the risk–return characteristics of different styles of assets in the securities market. Another reason is that in recent years, SW style indices have attracted much attention, and many fund products were designed with the SW style indices as a reference in the early stage of design. Since the publication of the SW style indices began on 30 December 1999, our research was calculated from the date of publication of the SW style indices, and the deadline for data recording was 30 December 2021. The data-recording period in this paper is long enough, with a total of 5333 trading days, including many influential periods in the Chinese stock market.

**Figure 2:**
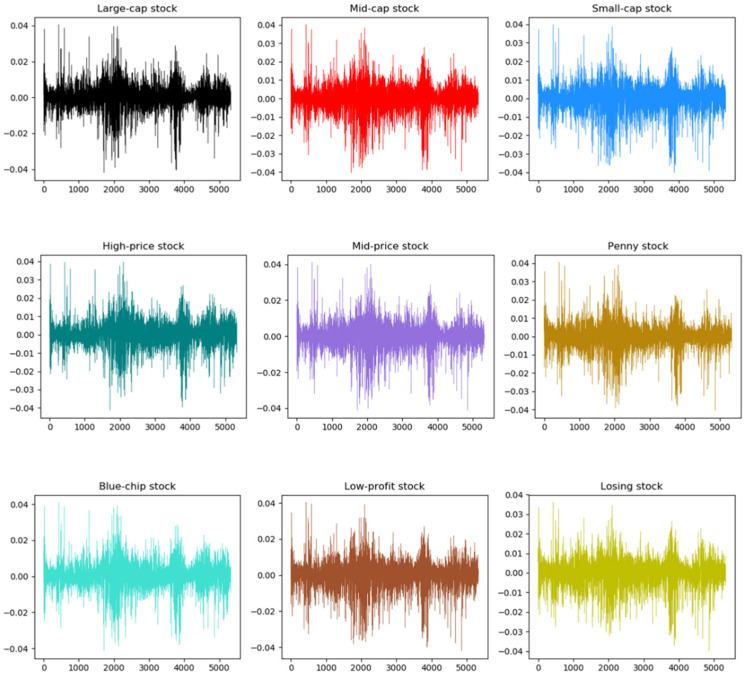
Return volatility of style stock indices.

We denote $p_{t}$ as the daily closing price of the style index at time t. In order to eliminate the possible heteroskedasticity of financial time series, we logarithmically process the original price series, as shown in the following formula. Figure 2 shows the volatility of the daily return series of the three types of style indices.

(15) $$r_{t}=log\;p_{t}-log\;p_{t-1}\;$$

We conducted a descriptive statistical analysis on the return series of various style indices, and the statistical results are recorded in Table 2. For the mean, there is no significant difference in the mean of each style index. Small-cap stock, penny stock and blue-chip stock average slightly higher than other style indices. From the standard deviation, it can be found that the volatility of small-cap stock in the style index related to the equity scale is relatively larger, the volatility of mid-price stock in the style index related to the share price is relatively larger and the volatility of low-profit stock in the style index related to the performance is relatively larger. The results of the kurtosis show that the kurtosis of the style indices is much greater than 3, and the kurtosis of the large-cap stock, the low-price stock and the blue-chip stock index is relatively larger. The skewness results reveal that all kinds of style index series are left-skewed. Compared with the standard normal distribution, all kinds of style indices in the stock market show the characteristics of left bias and sharp peaks. The results of the JB statistic imply that the style indices reject the assumption of normal distribution. The above statistical analysis shows that the return series of China’s stock market style indices all have the characteristics of sharp peaks and thick tails that financial time series usually have. Moreover, it can be found that there is a significant phenomenon of fluctuation aggregation in the stock market by the return sequence diagram. The phenomenon of volatility aggregation is also a manifestation of market inefficiency. This means that the return series of the stock market is not a random walk, but has the performance of trend continuation.

## 5. Results and Analysis of Style Stock Indices

### 5.1. Asymmetric Multifractal Analysis

**Figure 3:**
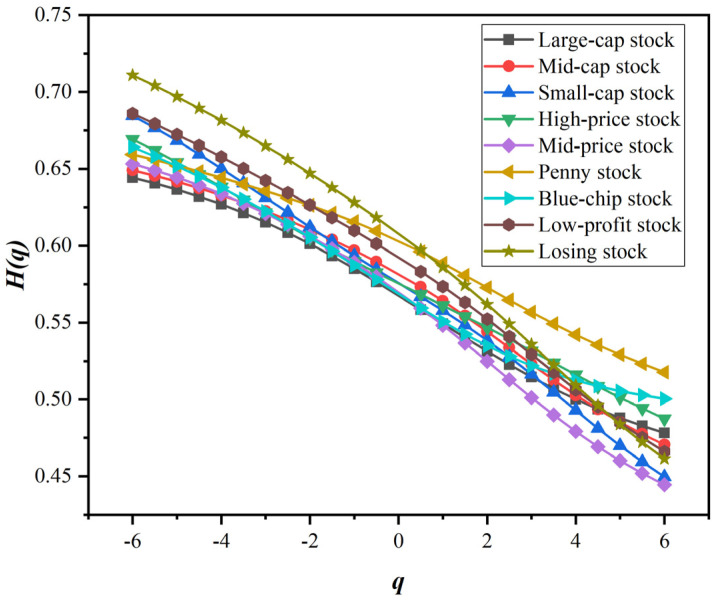
q−$H\left(q\right)$ of style stock indices.

Before using A-MFDFA or its extension method to explore the fractal characteristics of stock market, we first use MFDFA to test whether the fractal characteristics of style stock indices are single-fractal or multifractal. We plotted the generalized Hurst exponential curve with fluctuation order q by the MFDFA, as shown in Figure 3. The value range of the fluctuation order q is $\left(-6,6\right)$, which is used to measure the fluctuation range. It can be seen from the calculation steps of MFDFA that when q is large, it is the large wave component that plays a major role in the wave function $F_{q}\left(n\right)$. Therefore, when q is large, $H\left(q\right)$ mainly describes the fractal characteristics of large fluctuations. Similarly, when q is small, $H\left(q\right)$ describes the fractal characteristics of small fluctuations. However, when q is equal to 2, $H\left(2\right)$ is the classical Hurst index, which measures the overall long memory of the sequence.

Figure 3 shows that the Hurst values of the series of various style indices deviate significantly from 0.5, and the Hurst values of various style indices are significantly different. This proves that the style indices of the stock market have fractal characteristics and do not conform to the EMH under ideal conditions. The generalized Hurst values of the return series of the style indices are not invariable. The $H\left(q\right)$ of various style indices are not on the same horizontal line, and $H\left(q\right)$ changes significantly with the change in the fluctuation order q. This means that style indices do not show simple single-fractal characteristics, but have obvious multifractal characteristics. It is worth noting that with the increase in the fluctuation order q, the $H\left(q\right)$ of the style indices generally shows a monotonically decreasing trend. When q is small, the Hurst index of the return series of the style indices is often greater than 0.5, which shows that small fluctuations are often accompanied by strong persistence and the momentum of the fluctuation trend continues. For example, in a chronic bull market, stock prices do not rise suddenly, but in small fluctuations intermittently. Continued small moves contributed to the hold of the trend and the continuation of the bull market. On the other hand, when q is large, the Hurst index of the return series of the style stock indices is often less than 0.5. This shows that large fluctuations tend to have strong antipersistence, and the probability of reversal of the fluctuation trend increases. In times of sharp ups and downs, it is highly likely that the market is about to turn around and move in the opposite direction. In particular, when q is equal to 2, the $H\left(2\right)$ of each style stock index is also significantly higher than 0.5. This shows that the return series of the style stock indices have the characteristics of long memory. The price movement direction of the previous moment may carry over to the next moment.

In terms of the types of style indices, there are also some novel findings. The $H\left(q\right)$ of performance-related style indices are slightly higher than other types of style indices. Among the performance-type style indices, the $H\left(q\right)$ of the losing stock is the largest, followed by the low-profit stock, and the $H\left(q\right)$ of the blue-chip stock is relatively lower. However, among the style indices related to share price, the overall $H\left(q\right)$ of penny stock is higher than that of high-price stock and mid-price stock. In particular, the $H\left(q\right)$ of penny stock is always greater than 0.5 regardless of the value of q. The volatility persistence of penny stock is significantly stronger than that of other style indices. However, the difference about $H\left(q\right)$ in equity scale style indices is small. Overall, $H\left(q\right)$ of mid-cap stock and small-cap stock is slightly higher than that of large-cap stock. This is quite meaningful for investors, who can choose appropriate style assets for investment portfolios according to their own risk appetite.

**Figure 4:**
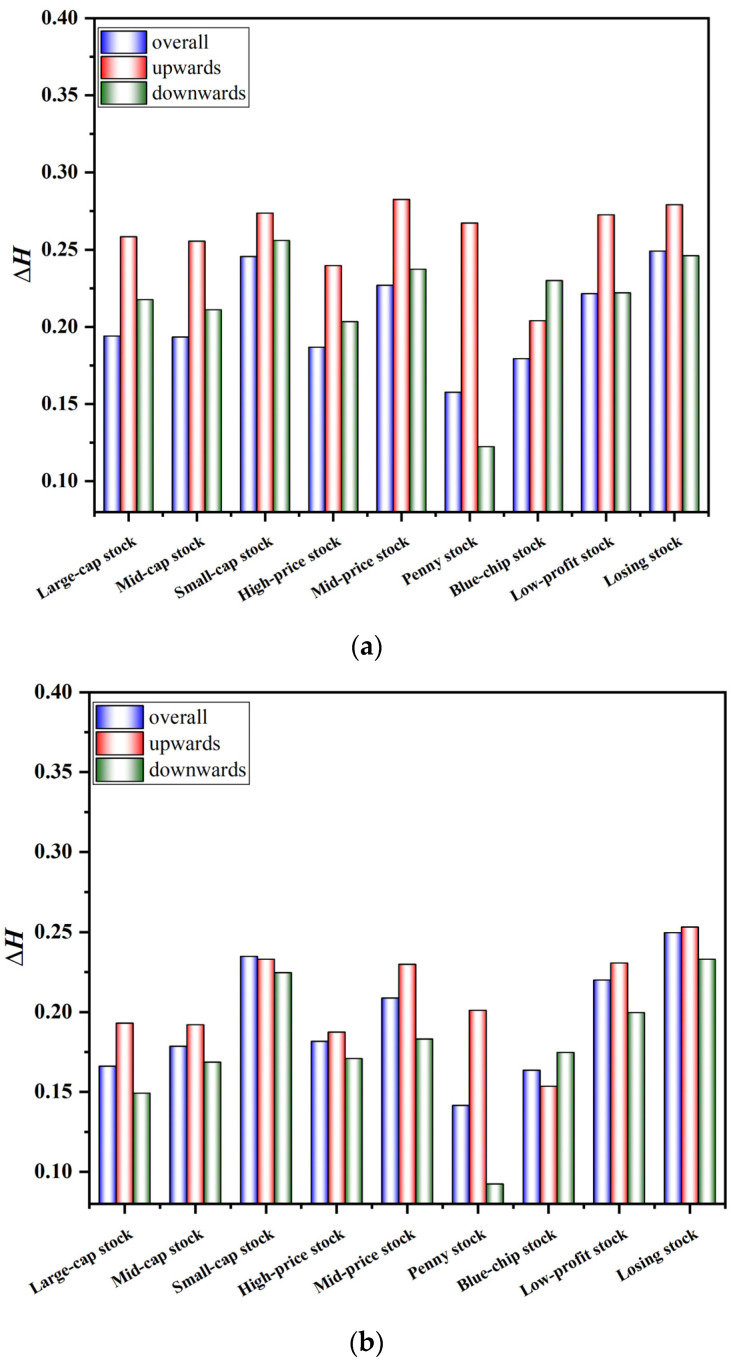
Computational differences between A−MFDFA and OSW−A−MFDFA. (**a**) Application Results of the A−MFDFA. (**b**) Application Results of the OSW−A−MFDFA.

The above research shows that it is an indisputable fact that the style indices exhibit multifractal characteristics. Investors are naturally curious about whether the fractal characteristics of the stock market under the upward trend and the downward trend are consistent. The proposed A-MFDFA allows us to explore multifractality under different trends. Theoretically, the discontinuity of the original A-MFDFA at the junctions of the split data may generate new pseudo-fluctuation errors, which in turn cause distortions in the scale index estimates. In view of its shortcomings, this paper uses the overlapping sliding window technology to improve and proposes OSW-A-MFDFA with more robust calculation results. In order to observe the calculation difference between A-MFDFA and OSW-A-MFDFA more clearly, we calculated the difference between the maximum value and the minimum value of $H\left(q\right)$ under different fluctuation orders q, and recorded it as ΔH. Specific results are provided in Figure 4. It is obvious that the ΔH under A-MFDFA application is significantly larger than that under OSW-A-MFDFA application. Compared with the original A-MFDFA, the Hurst exponential curve calculated by OSW-A-MFDFA is smoother. This shows that the improved algorithm reduces the pseudo-fluctuation error caused by discontinuous data segmentation in the traditional algorithm. The above results indirectly indicate that OSW-A-MFDFA has higher accuracy and can describe the multifractal characteristics of signals more scientifically and effectively. Therefore, this paper applies OSW-A-MFDFA to accurately reveal the fractal characteristics of style stock indices.

**Figure 5:**
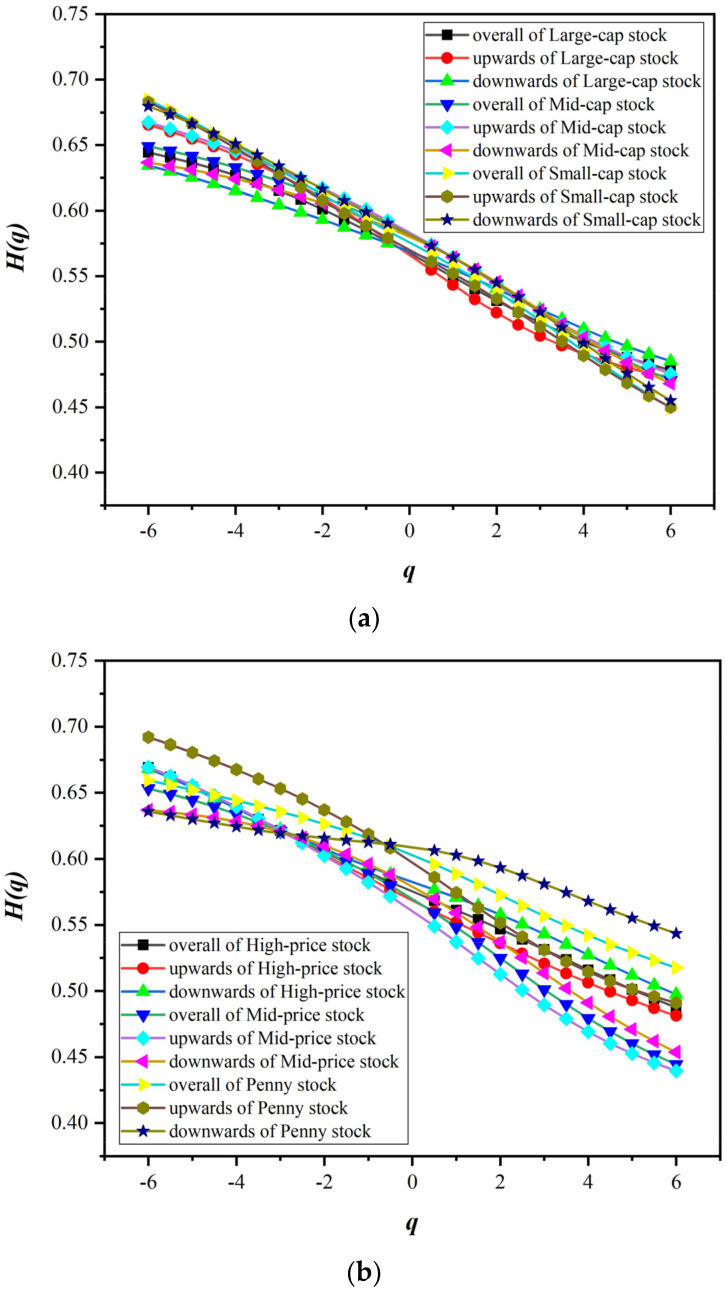

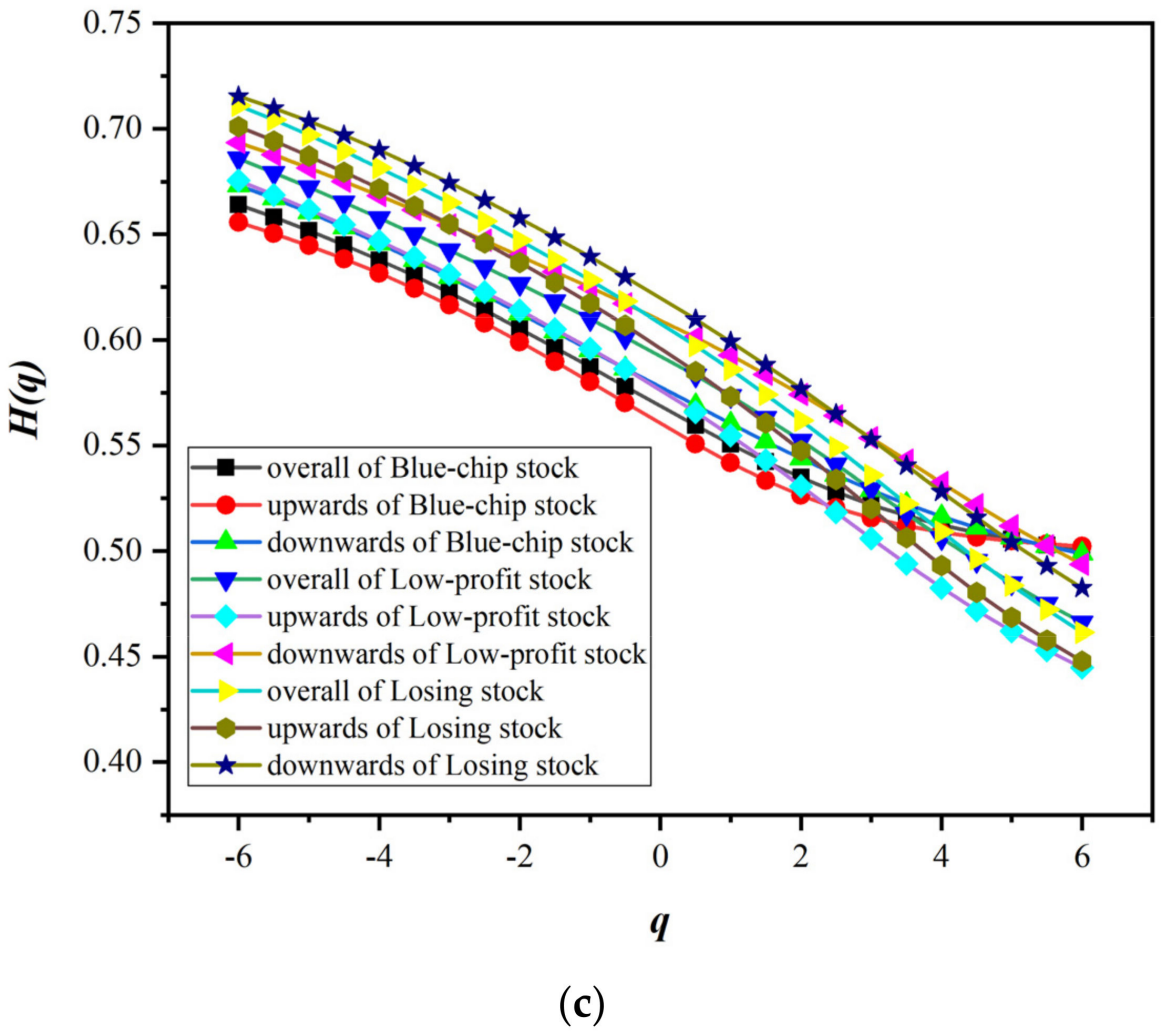
q−$H\left(q\right)$ of style stock indices under different trends. (**a**) Equity-scale-related style indices. (**b**) Stock−price−related style indices. (**c**) Performance−related style indices.

Next, we focus on the multifractal characteristics of style stock indices under different trends. Figure 5 shows the change in $H\left(q\right)$ corresponding to the fluctuation order q under different trends. It is obvious that there are differences in $H\left(q\right)$ at different scales and under different trends, which confirms that style stock indices have multifractal characteristics regardless of market trends. More specifically, no matter what kind of style index it is, the $H^{-}\left(q\right)$ under the downtrend is larger than the $H^{+}\left(q\right)$ under the uptrend, and the generalized Hurst value under the uptrend and downtrend is significantly different from the overall trend. This indirectly suggests that the stock market is less efficient on a downward trend. Therefore, the existing results favorably support the existence of asymmetric multifractal characteristics in the stock market. In addition, $H\left(q\right)$, $H^{+}\left(q\right)$ and $H^{-}\left(q\right)$ for all styles of stock indices decrease as q increases.

Specifically, the multifractal characteristics are asymmetric in the large-cap, mid-cap and small-cap indices with the equity scale as the distinguishing standard. For large-cap stock, when q is less than 0, $H^{+}\left(q\right)$ is greater than the $H\left(q\right)$ of the overall trend, and $H^{-}\left(q\right)$ is slightly lower. However, the opposite is true when q is greater than or equal to 0. Compared with $H^{+}\left(q\right)$ and $H\left(q\right)$, $H^{-}\left(q\right)$ is larger at this time. This shows that the fractal characteristics of large-cap stock are obviously different in the face of large fluctuations and small fluctuations. Under small fluctuations, large-cap stock has a stronger memory in the upward trend, while in the case of large fluctuations, large-cap stock is more likely to reverse under the upward trend. For mid-cap stock, when q is less than 0, $H^{+}\left(q\right)$ is at a higher level. When q is greater than or equal to 0, the Hurst values under different trends have little difference. Regardless of the value of q, small-cap stock exhibits $H^{-}\left(q\right)$ slightly higher than $H^{+}\left(q\right)$. Overall, the Hurst values for small-cap stock under different trends are not as varied as large-cap and mid-cap stocks.

Among the style indices related to share price, the degree of multifractality in the downtrend tends to be greater than that in the overall trend, and the degree of multifractality in the uptrend is the lowest. For high-price stock, when q is less than 0, the difference in Hurst values under the overall trend, uptrend and downtrend is less significant. However, when q is greater than or equal to 0, there are significant differences under different trends. $H^{-}\left(q\right)$ is higher than $H\left(q\right)$, and $H^{+}\left(q\right)$ is the lowest. When faced with large fluctuations, the stability of high-price stock decreases and the fluctuation increases. For penny stock, when q is less than 0, $H^{+}\left(q\right)$ is higher than $H^{-}\left(q\right)$. On the other hand, when q is greater than or equal to 0, the result is completely opposite, $H^{-}\left(q\right)$ is higher, and $H^{+}\left(q\right)$ is lower. Mid-price stock is similar to penny stock. The fractal characteristics of mid-price stock and penny stock under different scales are significantly different and less stable.

In the style indices related to performance, the generalized Hurst value of losing stock is higher than that of low-profit stock and blue-chip stock. Regardless of the value of the lag order q, the $H^{-}\left(q\right)$ of blue-chip stock, low-profit stock and losing stock in the downtrend is greater than $H\left(q\right)$, and $H^{+}\left(q\right)$ in the uptrend is the lowest. It is worth noting that the $H\left(q\right)$, $H^{+}\left(q\right)$ and $H^{-}\left(q\right)$ of blue-chip stock under different volatility orders q are all greater than 0.5, which indicates that the fluctuations in blue-chip stock are more persistent. However, when q is large, the generalized Hurst value of low-profit stock and losing stock will be lower than 0.5. This shows that the low-profit stock and the losing stock are more likely to change the volatility trend under large fluctuations. Overall, the volatility of blue-chip stock is more stable. This is similar to “The argosy cannot make a swift u-turn”.

It is worth noting that Lux (2004) has shown that typical “scale estimators” used in physics literature cannot distinguish between pseudo-multiscale and true multiscale of financial data, which may result in unreliable recognition of multifractal structures [53]. Furthermore, Lux (2004) mentions in the literature that in the presence of true multiscale, we would expect significant differences between original series and the shuffled series.

**Figure 6:**
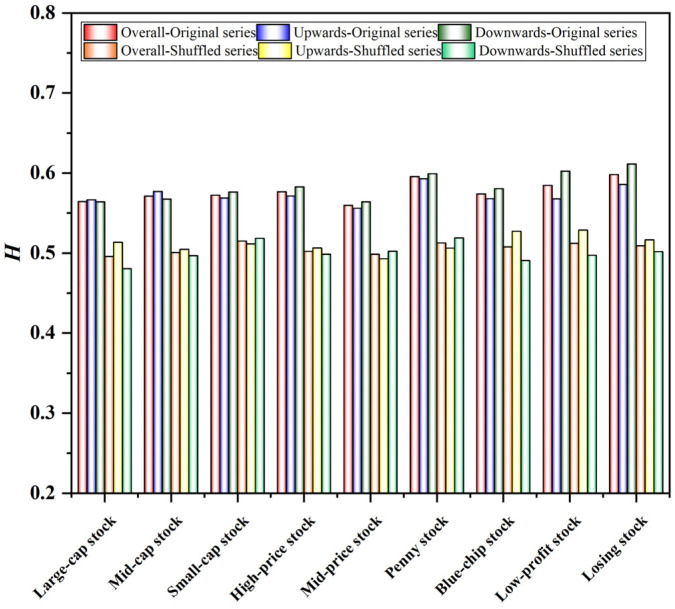
Differences between the original series and the shuffled series of the style stock indices.

We tested the effectiveness of the OSW-AMFDFA method used in this paper according to Lux’s exposition. The approach in this article is similar to that of Lux. We randomly shuffled the data of the return series of the style stock indices into the shuffled series. Then, we performed calculations separately for the original series and the shuffled series. The results show a significant difference in the Hurst index of the original and shuffled series, as shown in Figure 6. According to Lux’s research, there should be a significant difference between the original series and the shuffled series in the presence of true multiscale. Such results indirectly indicate that the calculation results of OSW-A-MFDFA in this paper are relatively reliable and robust.

In addition, the difference in Hurst values between the original series and the shuffled series of the style stock indices also reveals the source of the multifractal characteristics of the style stock indices. The shuffled series preserves the distribution of the series but destroys the correlation. That is, long memory is eliminated and the non-normality of sequence distribution is weakened. The Hurst value of the shuffled series is significantly lower than that of the original series, which indicates that the fractal characteristics of the style stock indices are related to the correlation of the possible price series.

**Figure 7:**
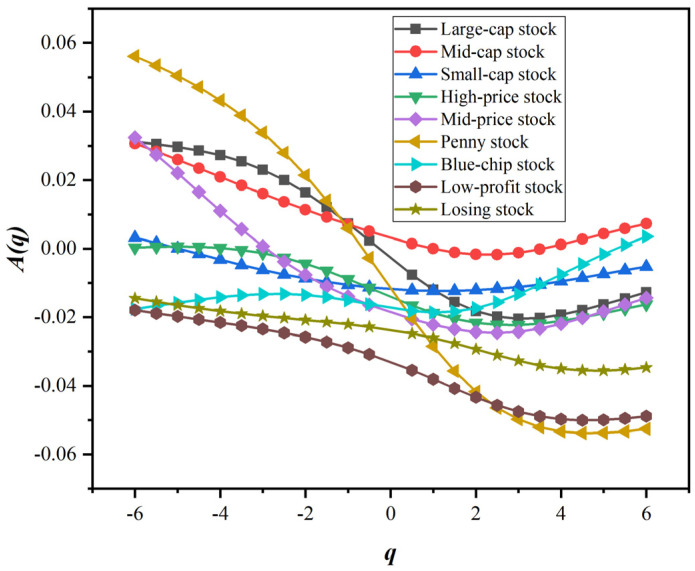
The degree of asymmetry of the fractal characteristics of style stock indices.

Next, we quantified the degree of asymmetry in the fractal characteristics of style stock indices, as shown in Figure 7. Specifically, in the style indices related to the equity scale, the degree of fractal asymmetry of small-cap stock is significantly smaller than that of large-cap and mid-cap stocks. In contrast, large-cap stock has the highest degree of fractal asymmetry. For style indices related to stock price, high-price stock has the least fractal asymmetry, followed by mid-price stock, and penny stock has the highest degree of asymmetry. In the performance-type style indices, the fractal asymmetry of blue-chip stock is significantly smaller than that of low-profit stock and losing stock. The degree of asymmetry of fluctuation correlation measures the difference of fluctuation correlation between uptrend and downtrend in return series. Theoretically, the lower the degree of asymmetry, the more stable the stock price volatility is. Corresponding to the style indices in this paper, large-cap stock, low-price stock and losing stock have a higher degree of asymmetry on the whole, and their fluctuations are more different under the upward trend and the downward trend. In particular, people tend to pay more attention to the asymmetric difference when q is equal to 2. In contrast, when q is equal to 2, the fractal characteristics of low-profit stock, losing stock and penny stock are more asymmetrical. Moreover, the $A\left(2\right)$ of various style stock indices are all negative, which means that the $H^{+}\left(2\right)$ of style stock indices is often smaller than $H^{-}\left(2\right)$. Furthermore, such results show that the long memory of the return series of the style stock indices is stronger in the downtrend than in the uptrend.

By applying the OSW-A-MFDFA to style stock indices, our findings are mainly as follows. The return series of the style indices have multifractal characteristics instead of single fractal. Furthermore, the multifractal characteristics of the style stock indices are asymmetrical, that is, the multifractal characteristics of the uptrend and the downtrend are significantly different. In addition, there are obvious differences in the fractal characteristics between different types of style indices.

It is worth mentioning that recognizing the asymmetry of multifractal characteristics is of great value to investors and regulators. For investors, recognizing the difference in the multifractal characteristics of the uptrend and the downtrend will help them adjust their investment strategies in a timely manner in the face of different trends. Investors expect higher returns and lower risk under different volatility trends. At the same time, the fractal characteristics of the style stock indices also help investors to further optimize their investment portfolios. Likewise, findings on asymmetric fractal characteristics are also valuable for regulators. Under different trends, regulators can focus on supervising assets that may change their volatility trends in the future, and take relevant measures to avoid abnormal market fluctuations. On the other hand, regulators can also intervene in a timely manner when the market is inefficient to ensure the healthy and sustainable development of the financial market.

### 5.2. Variation Analysis of Multifractal Spectrum

**Figure 8:**
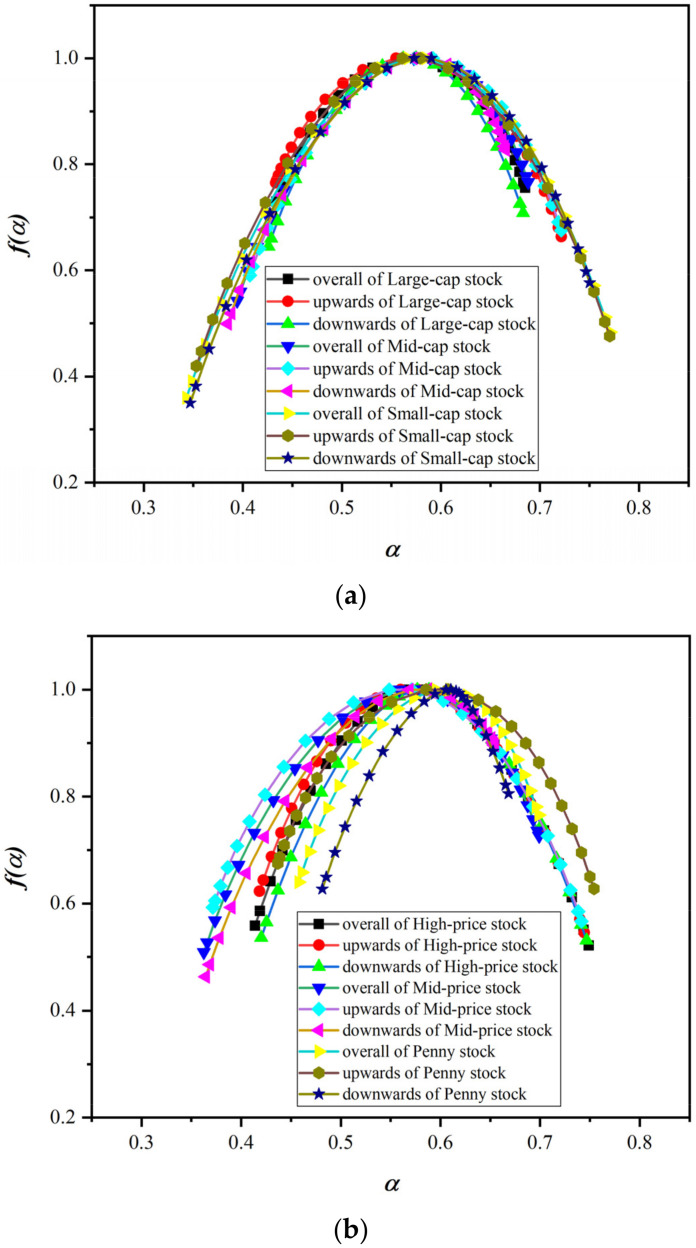

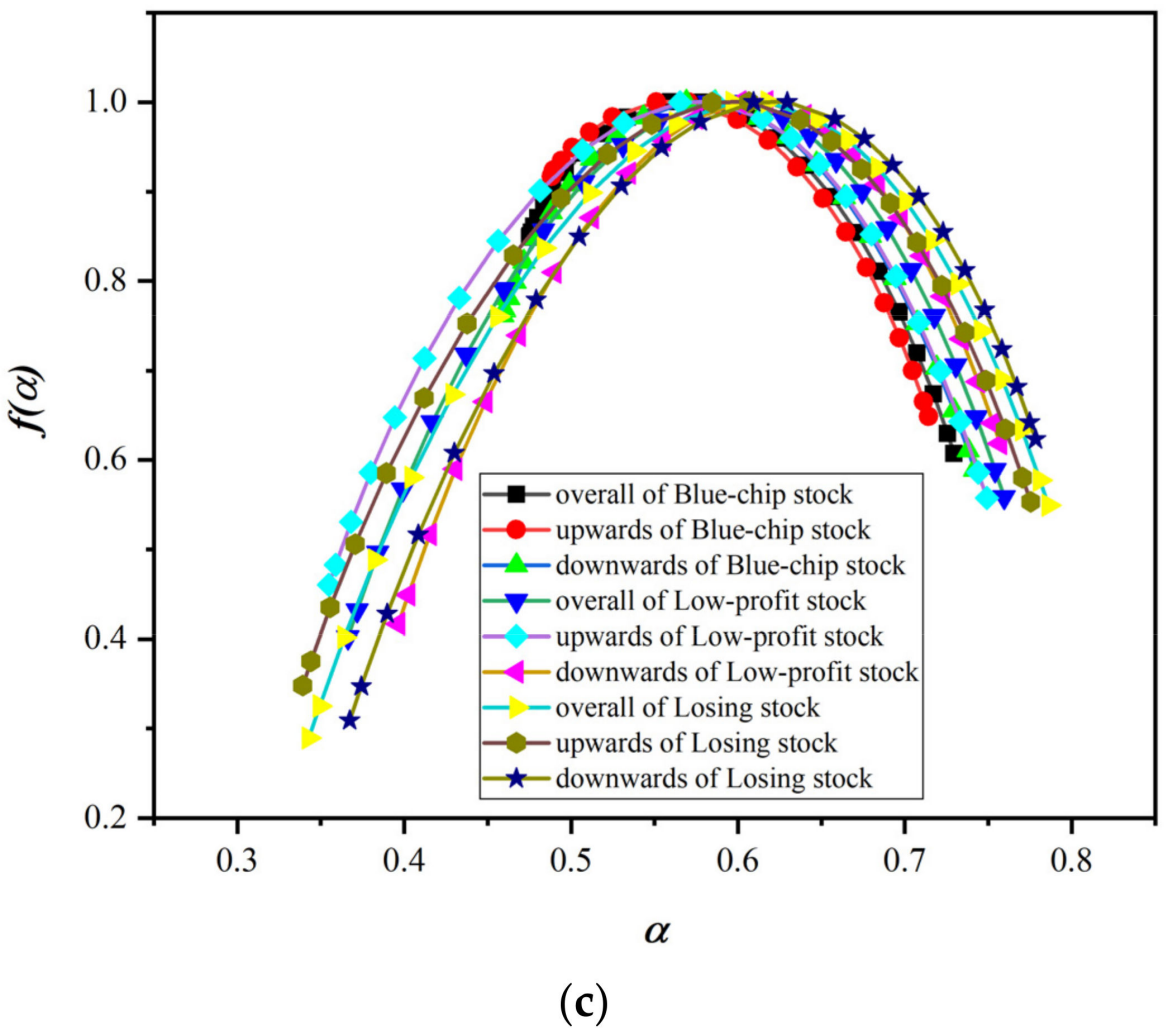
Multifractal spectrum of style stock indices. (**a**) Equity-scale-related style indices. (**b**) Stock-price-related style indices. (**c**) Performance-related style indices.

The multifractal spectrum can describe and measure the change process, complexity and inhomogeneity of the fractal structure. We plot the multifractal spectrum of the return series of the style indices, as shown in Figure 8. The width difference and height difference in the multifractal spectrum can characterize the fluctuation characteristics of the time series. The width difference dα of the multifractal spectrum characterizes the fluctuation and stability of the distribution of the entire time series after standard normalization. The height difference df of the multifractal spectrum represents the number of occurrences of the calculated singular values. Table 3, Table 4 and Table 5 provide important parameters of the multifractal spectrum of the style stock indices.

For the style indices related to the equity scale, the width difference dα of the multifractal spectrum of small-cap stock is significantly larger than that of large-cap stock and mid-cap stock, and the width difference dα of large-cap stock is the smallest. This shows that the price fluctuations of small-cap stock are relatively violent, and the price fluctuations of large-cap stock are relatively stable. For the height difference df of the fractal spectrum, df of large-cap stock is usually higher than df of small-cap stock, while df of mid-cap stock is smaller. The height difference df of the multifractal spectrum is positive only when large-cap stock is in an upward trend, and the df is negative in all other cases. This shows that the price sequence of large-cap stock appears in the trough position significantly less than that of mid-cap stock and small-cap stock during the fluctuation process. The style indices of the equity scale type have the smallest fractal spectrum width difference dα during the downtrend. For the df of the fractal spectrum under different trends, the df of large-cap, mid-cap and small-cap stocks is the smallest in the downtrend, and the largest in the uptrend.

For style indices related to stock price, regardless of the trend, the width difference dα of the multifractal spectrum of high-price and mid-price stocks is significantly larger than that of penny stock. Judging from the height difference df of the fractal spectrum, the df of the fractal spectrum of high-price stock is significantly larger than that of mid-price stock and penny stock. In particular, the df of the fractal spectrum of high-price stock is positive regardless of whether it is an overall trend or an upward or downward trend. This shows that the return series of high-price stock are in the peak of the wave more times in the fluctuation process. However, the df between mid-price stock and penny stock is positive during an uptrend, and negative during an overall trend and a downtrend. Overall, the dα and df of multifractal spectrum of high-price stock are larger. This means that although the volatility of high-price stock is also violent, the peaks are frequent, and risks and rewards coexist.

For performance-related style indices, the width difference dα of the multifractal spectrum of losing stock is the largest, followed by the dα of low-profit stock, and the dα of blue-chip stock is lower than the former two. This shows that the price volatility of losing stock is more volatile than that of low-profit and blue-chip stocks. The height differences df of the fractal spectrum of the blue-chip stock are all greater than 0, which indicates that the price of blue-chip stock is more frequently at the peak than at the trough. The height difference df of the multifractal spectrum of the low-profit stock and losing stock is less than 0, which indicates that they are in the trough more times in the process of price fluctuation. For different trends, blue-chip stock has the largest dα under the downward trend, while low-profit stock has the largest dα under the upward trend, and losing stock has the largest dα under the overall trend. All performance-related style indices have the largest fractal spectrum df under the uptrend, and the smallest df under the downtrend.

### 5.3. Efficiency Analysis of Style Indices

The above discussion has confirmed the existence of asymmetric multifractal characteristics in the stock market. Considering this realistic situation, the scientific nature of measuring market efficiency is worth discussing. The generalized Hurst index contains a large amount of market information and can be used to characterize market efficiency. It is well known that the Hurst index of a sequence obeying a random walk in an efficient market is 0.5. However, if the Hurst index of the stock market’s return series deviates significantly from 0.5, it indicates that the stock price movement does not follow a random walk. Furthermore, this also means that the stock market is not a perfectly efficient market. If the Hurst index of the security return series is significantly greater than 0.5, it means that the security return series is likely to have long memory. The price movement trend in the last moment is likely to continue at the current moment. Based on this, this paper constructs the market efficiency index MED by combining the characteristics of multifractal. The advantage of the MED indicator is that it traverses all the fluctuation orders q and fully considers the existence of multifractals.

**Figure 9:**
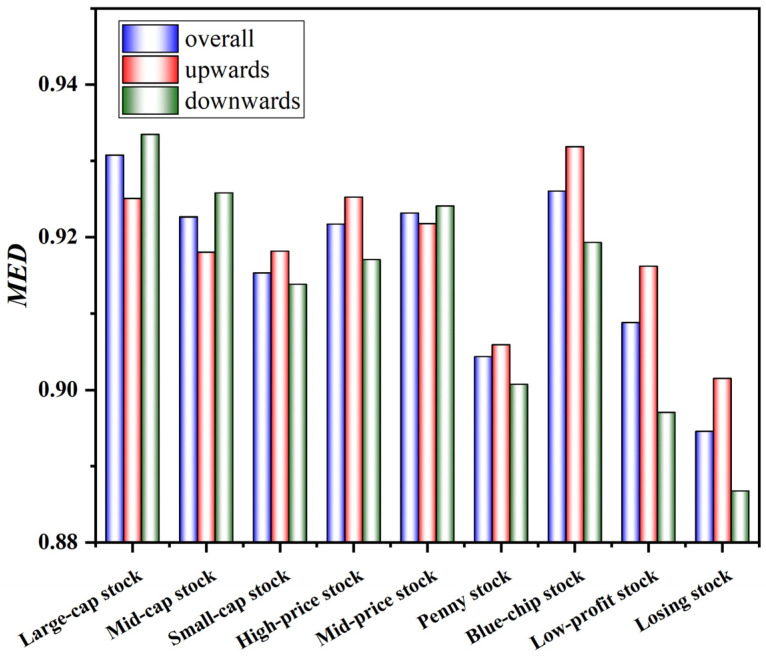
Market efficiency of style indices.

Figure 9 provides the degree of market efficiency for style indices. Whether in the overall trend, the upward trend or downward trend, the market efficiency of large-cap stock is higher than that of mid-cap stock and small-cap stock, the market efficiency of high-price stock and mid-price stock is much higher than that of penny stock, and the market efficiency of blue-chip stock is higher than that of low-profit stock and losing stock. However, it is not enough to analyze the overall market efficiency. We are naturally curious about whether the market efficiency of the stock market will change over time.

**Figure 10:**
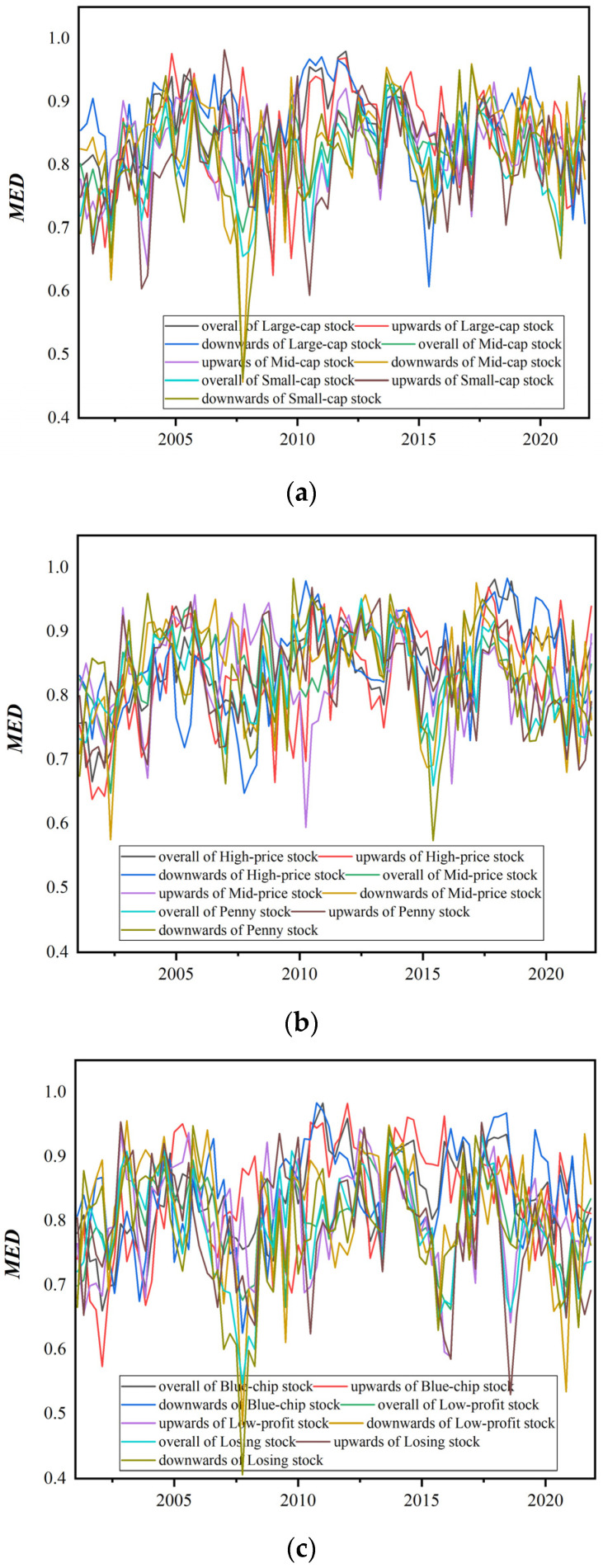
Time-varying market efficiency of style indices. (**a**) Equity-scale-related style indices. (**b**) Stock-price-related style indices. (**c**) Performance−related style indices.

Next, we present the market efficiency in different periods through the MED indicator, and further demonstrate the dynamic evolution process of market efficiency from a local perspective, and then study the adaptive characteristics of the stock market. Specifically, the technique of overlapping sliding windows is used to asymptotically analyze the degree of market efficiency of style stock indices. Since the length of the data sample in this paper is as long as 20 years, we set the length of the window period to 240 working days (about the length of one year), and the sliding step size to 60 working days (about the length of a quarter). The market efficiency degree of each group of subsamples is calculated separately, and the time-varying market efficiency degree of the style indices is finally presented, as shown in Figure 10.

Figure 10 shows that the degree of market efficiency of the stock market style indices varies significantly over time, regardless of market trends. The line graph of market efficiency goes from low to high, and then from high to low, with peaks and troughs. The market efficiency of all style indices is volatile and cyclical. From a local point of view, the market efficiency of style indices has alternate cycles of effectiveness and ineffectiveness, indicating that the effectiveness of market efficiency is a time-varying process. In addition, the peaks and troughs of the market efficiency curves of different style indices are similar, which also shows that the volatility between stock markets is synergistic.

In recent years, the adaptive market hypothesis (AMH) has been in the ascendant, which can reasonably explain this phenomenon [54]. The AMH believes that market efficiency is not stable, and its effectiveness and ineffectiveness are the performance of adaptive characteristics, and long-term market efficiency and stability cannot be guaranteed [55]. Changes in conditions such as the stability of the overall market environment, the number of competitors, available profit opportunities and the adaptability of market participants will lead to constant changes in market efficiency [56,57]. When there are major changes, such as political, economic, cultural and social changes that affect the market environment, the price of the stock market changes immediately.

In contrast, we are more concerned about the moments of low market efficiency, because a sustained low market efficiency may bring incalculable risks to investors. Figure 10 shows that the market efficiency curves for style indices have exhibited several periods of persistent inefficiency over the past two decades. Firstly, the market efficiency in 2001–2002 was in a state of low efficiency. This may be related to the fact that the Chinese government issued regulations on the reduction in state-owned shares in 2001, and the SARS epidemic in 2002, which weakened the effectiveness of the stock market. After investors gradually adapted to the environment, the market efficiency rebounded. After that, the market efficiency curves of the style indices were in a trough position in 2007–2009. This is mainly attributable to the impact of the financial crisis. The subprime mortgage crisis in the United States in 2007, which in turn triggered the global financial crisis in 2008, and caused the stock market to plummet in effectiveness. However, the persistent inefficiency led the government to take relevant measures to rescue the market, and the stock market gradually became more effective after adapting to the major changes in this period. The next wave of persistent inefficiencies in the stock market was concentrated in 2014–2015, but market efficiency gradually picked up as market participants adapted. The most recent period of continued market inefficiency is mainly concentrated in 2020–2021. This stage coincides with the ravages of COVID-19 around the world, which has brought many negative shocks to financial markets, making the stock market relatively inefficient.

Evidence under different trends shows that the market efficiency of the stock market has time-varying characteristics, and the market efficiency will change dynamically with time and interaction environment. This confirms the view of the AMH. The sudden change in the market environment will have an impact on market participants, and it will take a certain amount of time to adapt, and the efficiency of the market will decrease in stages. When market participants gradually adapt to the mutation, the efficiency of the market will also increase. The time-varying and cyclical nature of market efficiency indicates that the stock market is an evolving process of continuous adaptation. Faced with the dynamic evolution of market efficiency, market participants should also adjust their investment strategies in a timely manner.

## 6. Conclusions

As a barometer of the financial market, the volatility characteristics of the stock market have always been the focus of the academic and practical circles. It is an indisputable fact that the stock market has fractal nonlinear characteristics. It is imperative to fully understand the fractal characteristics of the stock market and reasonably analyze the market efficiency. However, previous studies have mainly focused on the composite indices of the stock market, and few studies have focused on the style indices. Style index is a type of index that reflects a particular style or investment characteristics in the market. On the one hand, the style index can better reflect the risk–return characteristics of different styles of assets in the securities market. On the other hand, the design of many fund products is often based on the style index. In view of the important value of style indices, this paper deeply analyzes the fractal characteristics of different types of style indices.

In recent years, DFA and MFDFA methods have played an important role in fractal research. Such methods can effectively exclude spurious long-range correlations caused by the nonstationarity of time series, thereby truly revealing the part of long-range correlations that characterize the dynamic behavior of complex systems. Unfortunately, the MFDFA cannot reveal the fractal characteristics under different trends, while the A-MFDFA can additionally explore the fractal characteristics under different trends while retaining the advantages of MFDFA. In this paper, the A-MFDFA is further improved, and the overlapping sliding window technique is combined with the original A-MFDFA to construct OSW-A-MFDFA with more robust and reliable calculation results. Furthermore, this paper pays attention to the changes in the width difference and height difference of the multifractal spectrum under the application of OSW-A-MFDFA. In addition, this paper proposes a market efficiency measure MED that can traverse all volatility orders q. Its advantage is that it fully considers the reality of multifractality, and can more reasonably analyze the effectiveness of the stock market.

Taking the style indices related to equity scale, share price and performance as a research sample, we obtained some interesting findings. Firstly, the style stock indices have multifractal characteristics, and the fractal characteristics are asymmetric. There is a significant difference in the multifractal characteristics of the uptrend and the downtrend, and the fractal degree of the downtrend is often stronger than that of the uptrend. Different types of style indices also have different multifractal characteristics. This paper further quantifies the degree of asymmetry of the multifractal characteristics, and we found that the fractal characteristics of blue-chip stock, high-price stock and small-cap stock have a lower degree of asymmetry. Secondly, the multifractal spectrum of different types of style indices also conveys many market volatility information. The width difference and height difference of the multifractal spectrum under different market trends are also significantly different. The width difference in the fractal spectrum of losing stock, high-price stock and small-cap stock is relatively larger. The height difference in the fractal spectrum of large-cap stock, blue-chip stock and high-price stock is relatively larger. Thirdly, with the help of the MED index to analyze the effectiveness of the style stock indices, we found that the market effectiveness of the style index has time-varying characteristics. The degree of market efficiency in different periods changes dynamically, and the effectiveness of the stock market in crisis periods is in a continuous downturn. The AMH can reasonably explain such results. The sudden change in the market environment during the crisis period will have an impact on market participants, and it will take a certain amount of time to adapt, and the efficiency of the market will decrease in stages. When market participants gradually adapt to the mutation, the efficiency of the market will gradually increase.

It is worth noting that we should also clearly recognize that various multifractal models still have problems such as difficult modeling, no clear standard for algorithm selection and complexity of estimation methods. In the future, scholars still need to continue to optimize the model. It is expected that the findings of this paper on the fractal characteristics of style indices can be further applied to practical fields, such as trend forecasting in the stock market and optimization of investment portfolios. The research in this paper has not been combined with investment strategy. We certainly hope to further improve the returns of investment strategies and reduce risks through the analysis of fractal characteristics, which is also the direction of our future efforts. In addition, it is foreseeable that the OSW-A-MFDFA method and MED index in this paper are also applicable to other aspects of physics and informatics to reveal more novel discoveries.

## Figures and Tables

**Table 1:** The research sample.

Factor	Style Stock Indices		
Equity scale	Large-cap stock	Mid-cap stock	Small-cap stock
Share price	High-price stock	Mid-price stock	Penny stock
Performance	Blue-chip stock	Low-profit stock	Losing stock

**Table 2:** Descriptive statistics results of style stock indices.

	Mean	Maximum	Minimum	Std. Dev	Kurtosis	Skewness	Jarque–Bera
Large-cap stock	1.20 × 10^−4^	0.0407	−0.0420	0.0070	7.6066	−0.3184	4.80 × 10^3^
Mid-cap stock	1.30 × 10^−4^	0.0403	−0.0404	0.0076	6.7972	−0.6361	3.56 × 10^3^
Small-cap stock	1.40 × 10^−4^	0.0399	−0.0404	0.0078	6.5100	−0.6856	3.15 × 10^3^
High-price stock	1.30 × 10^−4^	0.0396	−0.0413	0.0072	6.4285	−0.2931	2.69 × 10^3^
Mid-price stock	1.00 × 10^−4^	0.0412	−0.0412	0.0077	6.9325	−0.5440	3.70 × 10^3^
Penny stock	2.00 × 10^−4^	0.0405	−0.0403	0.0071	7.5415	−0.5761	4.88 × 10^3^
Blue-chip stock	1.80 × 10^−4^	0.0410	−0.0415	0.0074	6.7421	−0.2665	3.17 × 10^3^
Low-profit stock	8.00 × 10^−5^	0.0404	−0.0421	0.0082	6.2889	−0.7054	2.85 × 10^3^
Losing stock	1.00 × 10^−4^	0.0361	−0.0399	0.0074	5.7018	−0.6924	2.05 × 10^3^

**Table 3:** Fractal spectrum parameter statistics for style indices of equity scale.

	$\alpha_{min}$	$\alpha_{max}$	dα	$f\left(\alpha_{min}\right)$	$f\left(\alpha_{max}\right)$	df
Overall of large-cap stock	0.4277	0.6850	0.2573	0.6966	0.7558	−0.0592
Upwards of large-cap stock	0.4334	0.7217	0.2883	0.7651	0.6632	0.1019
Downwards of large-cap stock	0.4260	0.6829	0.2570	0.6447	0.7083	−0.0635
Overall of mid-cap stock	0.3943	0.6880	0.2937	0.5424	0.7669	−0.2242
Upwards of mid-cap stock	0.4072	0.7215	0.3143	0.5908	0.6753	−0.0846
Downwards of mid-cap stock	0.3846	0.6655	0.2808	0.4993	0.8272	−0.3279
Overall of small-cap stock	0.3430	0.7706	0.4276	0.3596	−0.4833	−0.1238
Upwards of small-cap stock	0.3532	0.7703	0.4171	0.4194	0.4758	−0.0564
Downwards of small-cap stock	0.3468	0.3498	0.4035	0.7503	0.5768	−0.2271

**Table 4:** Fractal spectrum parameter statistics for style indices of share price.

	$\alpha_{min}$	$\alpha_{max}$	dα	$f\left(\alpha_{min}\right)$	$f\left(\alpha_{max}\right)$	df
Overall of high-price stock	0.4139	0.7489	0.3349	0.5588	0.5216	0.0373
Upwards of high-price stock	0.4184	0.7444	0.3260	0.6230	0.5456	0.0774
Downwards of high-price stock	0.4202	0.7465	0.3263	0.5364	0.5311	0.0053
Overall of mid-price stock	0.3626	0.6989	0.3363	0.5087	0.7261	−0.2175
Upwards of mid-price stock	0.3716	0.7415	0.3699	0.5926	0.5670	0.0256
Downwards of mid-price stock	0.3645	0.4636	0.2899	0.6544	0.8952	−0.4316
Overall of penny stock	0.4578	0.6985	0.2407	0.6402	0.7655	−0.1253
Upwards of penny stock	0.4369	0.7541	0.3172	0.6754	0.6279	0.0475
Downwards of penny stock	0.4814	0.6683	0.1868	0.6274	0.8057	−0.1783

**Table 5:** Fractal spectrum parameter statistics for style indices of share performance.

	$\alpha_{min}$	$\alpha_{max}$	dα	$f\left(\alpha_{min}\right)$	$f\left(\alpha_{max}\right)$	df
Overall of blue-chip stock	0.4753	0.7295	0.2542	0.8490	0.6073	0.2416
Upwards of blue-chip stock	0.4896	0.7143	0.2247	0.9242	0.6486	0.2756
Downwards of blue-chip stock	0.4591	0.7418	0.2827	0.7621	0.5892	0.1729
Overall of low-profit stock	0.3663	0.7595	0.3932	0.4016	0.5588	−0.1572
Upwards of low-profit stock	0.3549	0.7492	0.3943	0.4603	0.5577	−0.0974
Downwards of low-profit stock	0.3965	0.7570	0.3605	0.4168	0.6185	−0.2017
Overall of losing stock	0.3429	0.7861	0.4431	0.2892	0.5493	−0.2601
Upwards of losing stock	0.3392	0.7754	0.4362	0.3480	0.5535	−0.2055
Downwards of losing stock	0.3673	0.7783	0.4109	0.3089	0.6229	−0.3141

## Data Availability

This paper analyzes publicly available datasets. Details can be found below: https://github.com/Crazy-xc/Asymmetric-multifractals accessed on 25 May 2022.
